# Measuring subjective stress among young people in Hong Kong: validation and predictive utility of the single-item subjective level of stress (SLS-1) in epidemiological and longitudinal community samples

**DOI:** 10.1017/S2045796021000445

**Published:** 2021-09-08

**Authors:** S. M. Y. Wong, B. Y. H. Lam, C. S. M. Wong, H. P. Y. Lee, G. H. Y. Wong, S. S. Y. Lui, K. T. Chan, M. T. H. Wong, S. K. W. Chan, W. C. Chang, E. H. M. Lee, Y. N. Suen, C. L. M. Hui, E. Y. H. Chen

**Affiliations:** 1 Department of Psychiatry, LKS Faculty of Medicine, University of Hong Kong, Pok Fu Lam, Hong Kong; 2 Department of Social Work and Social Administration, University of Hong Kong, Pok Fu Lam, Hong Kong; 3 Key Laboratory of Brain and Cognitive Sciences, University of Hong Kong, Pok Fu Lam, Hong Kong

**Keywords:** Adolescents, depression, epidemiology, stress, validation study

## Abstract

**Aims:**

Brief measurements of the subjective experience of stress with good predictive capability are important in a range of community mental health and research settings. The potential for large-scale implementation of such a measure for screening may facilitate early risk detection and intervention opportunities. Few such measures however have been developed and validated in epidemiological and longitudinal community samples. We designed a new single-item measure of the subjective level of stress (SLS-1) and tested its validity and ability to predict long-term mental health outcomes of up to 12 months through two separate studies.

**Methods:**

We first examined the content and face validity of the SLS-1 with a panel consisting of mental health experts and laypersons. Two studies were conducted to examine its validity and predictive utility. In study 1, we tested the convergent and divergent validity as well as incremental validity of the SLS-1 in a large epidemiological sample of young people in Hong Kong (*n* = 1445). In study 2, in a consecutively recruited longitudinal community sample of young people (*n* = 258), we first performed the same procedures as in study 1 to ensure replicability of the findings. We then examined in this longitudinal sample the utility of the SLS-1 in predicting long-term depressive, anxiety and stress outcomes assessed at 3 months and 6 months (*n* = 182) and at 12 months (*n* = 84).

**Results:**

The SLS-1 demonstrated good content and face validity. Findings from the two studies showed that SLS-1 was moderately to strongly correlated with a range of mental health outcomes, including depressive, anxiety, stress and distress symptoms. We also demonstrated its ability to explain the variance explained in symptoms beyond other known personal and psychological factors. Using the longitudinal sample in study 2, we further showed the significant predictive capability of the SLS-1 for long-term symptom outcomes for up to 12 months even when accounting for demographic characteristics.

**Conclusions:**

The findings altogether support the validity and predictive utility of the SLS-1 as a brief measure of stress with strong indications of both concurrent and long-term mental health outcomes. Given the value of brief measures of mental health risks at a population level, the SLS-1 may have potential for use as an early screening tool to inform early preventative intervention work.

## Background

Stress is one of the most ubiquitous experiences in daily life. Defined as the extent to which one considers one's life as stressful, the experience of stress could be conceptualised to be generated via dynamic interactions between internal states and the external environment (Epel *et al*., 2018).

Low levels of stress may help to increase alertness and enhance detection of danger and threat in the environment (Godoy *et al*., 2018). Nonetheless, excessive and chronic stress could result in dysfunctions in different cognitive and brain networks (Liston *et al*., 2009) as well as decompensations in internal physiological systems responsible for adaptations to the environment (e.g. the sympathetic–adrenal–medullary system and hypothalamic–pituitary–adrenal axis; Chrousos, 1998; McEwen, 2006). A state of allostatic overload may inflict adverse consequences on brain tissues via mechanisms such as the overproduction of glucocorticosteroids, excessive oxidative stress and heightened neuroinflammatory cytokine activities, which could in turn contribute to a range of detrimental long-term mental and physical health outcomes (Miller and Raison, 2016).

Stress can be measured both objectively (e.g. via physiological indicators, such as cortisol level and heart rate variability) and subjectively (e.g. using psychometric scales). Negative effects of the subjective experience of stress in response to external stressors on mental well-being have been documented. Studies have consistently shown that perceived stress is implicated in a wide range of psychiatric conditions, including depression, anxiety, post-traumatic stress disorder and psychotic disorders (Schneiderman *et al*., 2005; Barbui and Tansella, 2013). Stress has also been postulated to contribute to anhedonia (as an endophenotype of depression) and affect brain reward pathways (e.g. mesocorticolimbic dopaminergic systems), which could lead to dysregulations in reward-related decision making and reinforcement learning (Pizzagalli, 2014).

Monitoring the experience of stress during youth (between the ages of 15 and 24) is particularly important. This period not only marks a time of important life transitions, but also the maturation of different brain systems and functions, such as executive functioning, higher-order cognitive processing, self-regulation capabilities and social and emotional processing (Johnson *et al*., 2009). Reviews have shown that a wide range of mental disorders have their onset during youth that could persist into adulthood, including mood, anxiety, psychotic and substance use disorders (Kessler *et al*., 2007). The global burden of disease and long-term cost implications are therefore especially significant for this population (Erskine *et al*., 2015).

Elaborate measures would be ideal for assessing mental health conditions, yet their implementations may not always be feasible, particularly in epidemiological surveys, routine evaluation in services, and in time-limited and low-resource settings (Kessler *et al*., 2010). Although single-item ratings of stress have been utilised in work settings (e.g. Elo *et al*., 2003), there remains a lack of standardised simple measures of psychological stress in population mental health contexts. Simple measures of subjective stress in good agreement with other well-established symptom measures could facilitate large-scale screening of risk in community settings to reduce manpower burden and enhance cost-effectiveness of early detection and prevention (Mihalopoulos *et al*., 2011). A valid single stress measure would also be essential for the momentary assessment of stress in daily contexts (Myin-Germeys *et al*., 2018). We therefore developed an accessible and convenient measure to assess self-reported stress which we termed the single-item subjective level of stress (SLS-1).

We first determined the content and face validity of the SLS-1. Two studies were then conducted to assess the validity and predictive utility of the SLS-1 in separate samples of young people in Hong Kong. In study 1, in a large epidemiological sample, we examined the convergent and divergent validity as well as incremental validity of the SLS-1. In study 2, in addition to the above validation procedures, a longitudinal community sample was used to examine the relationship between baseline SLS-1 scores and long-term mental health outcomes of up to 12 months to test its predictive capability.

## Methods

### Participants and study settings

A panel was formed with eight mental health experts (psychiatrists and senior researchers of the project team) and 12 laypersons (young people not involved in the current study) to assess the content and face validity of the SLS-1 using an anonymous online questionnaire.

#### Study 1 (validation study)

Participants were recruited from the larger Hong Kong Youth Epidemiological Study of Mental Health, which is an ongoing territory-wide, household-based epidemiological study of mental disorders in a large representative sample of young people in Hong Kong. As in previous population research (Lam *et al*., 2015), the current study adopted a stratified multistage cluster sampling design to ensure sample representativeness. Randomly selected addresses, stratified by geographical location and type of housing quarters, were provided by the Census and Statistics Department of the Hong Kong Special Administrative Region. Invitation letters were sent to the selected addresses. All young people aged between 15 and 24 were included.

As part of the larger study, data on SLS-1 from 1695 consecutive participants were collected between May 2019 and January 2021 through face-to-face interviews (except during coronavirus disease 2019 [COVID-19] lockdown, online video interviews were conducted). Both participant-administered and researcher-rated measures were used to obtain information including sociodemographic and lifestyle characteristics, common and severe mental disorders, childhood experiences, life events, psychosocial functioning and service utilisation.

#### Study 2 (longitudinal outcome prediction study)

The longitudinal sample involving 266 consecutive participants was recruited as part of a larger ongoing community mental health project in collaboration with local youth centres in Hong Kong. The project aims to provide service to young people between the ages of 12 and 24. All service users of this project or other generic local youth services between 12 and 24 years were included in the study. Those with a known diagnosis of psychiatric disorder, currently receiving psychiatric medication, or with limited comprehension ability due to epilepsy or known intellectual disability were excluded. Participants were followed up and re-assessed at 3, 6 and 12 months (online Supplementary material Fig. S1). Aiming for 80% power in the longitudinal analyses, with a correlation coefficient of 0.30 (at the *p* = 0.05 significance level), a sample size of 85 or above was suggested. Baseline and follow-up data analysed in the current study were collected between December 2019 and March 2021.

Written informed consent was obtained from all participants. Consent was obtained from parents or legal guardians for those aged below 18 years. Ethical approval was obtained from the Institutional Review Board of the University of Hong Kong/Hospital Authority Hong Kong West Cluster. All participants were Cantonese speaking.

### Measures

Demographic information collected in both samples included age, gender and highest level of education (primary or below, secondary, tertiary or above).

The SLS-1 was assessed with the following instruction in Chinese: ‘Please indicate the level of stress which you consider you have experienced in the past one month on a scale of 0 to 10’ (on an 11-point Likert scale, where 0 = ‘not at all’, 10 = ‘extremely’, 5 = ‘moderate’).

Symptoms of psychological distress were assessed using the six-item Kessler Psychological Distress Scale (K6; Kessler *et al*., 2003), with each item rated on a 5-point Likert scale (from ‘all of the time’ to ‘none of the time’). The Chinese version of K6 has demonstrated good internal consistency (with a single factor explaining 59.8% of the total variance) and validity (receiver operating characteristics curve of 0.90 judged against the Beck Depression Inventory-II) in a youth sample in Hong Kong (Chan and Fung, 2014). Cronbach's alpha of K6 in samples 1 and 2 were 0.91 and 0.87, respectively.

The Depression Anxiety Stress Scale (DASS-21; Lovibond and Lovibond, 1995) contains three seven-item subscales assessing symptoms of depression (DASS-D), anxiety (DASS-A) and stress (DASS-S) in the past week rated on a 4-point Likert scale (from ‘did not apply to me’ to ‘applied to me very much’). The final subscale scores were computed by multiplying the summed items by a value of two. The DASS-21 has been validated in youth samples (Szabó, 2010) and has shown good internal consistency (*α* = 0.74–0.84) and convergent validity (*r* = −0.47–0.58 with the mental health score on the Adolescent Duke Health Profile measure; Le *et al*., 2017). The scale has also been validated in Chinese samples (Wang *et al*., 2016). Cronbach's alpha of the DASS-21 subscales was 0.81–0.88 and 0.84–0.89 in the two current samples, respectively.

The Patient Health Questionnaire-9 (PHQ-9; Kroenke *et al*., 2001) assesses depressive symptom severity during the past 2 weeks according to the nine symptoms in the DSM-IV on a 4-point Likert scale (from ‘not at all’ to ‘nearly every day’). Good internal consistency (*α* = 0.86) and convergent validity (*r* = 0.79 with anxiety symptoms, *r* = −0.60 with perceived control) have been reported in young people in Hong Kong (Leung *et al*., 2020). Internal consistency of the scale in the current studies was good (*α* = 0.89 and *α* = 0.90, respectively).

The General Anxiety Disorder-7 (GAD-7; Spitzer *et al*., 2006) assesses anxiety symptom severity during the past 2 weeks with seven items rated on a 4-point Likert scale (from ‘not at all’ to ‘nearly every day’). The GAD-7 has shown good internal consistency (*α* = 0.91) and convergent validity (*r* = 0.46 with the Mini-Social Phobia Inventory) in a youth sample (Tiirikainen *et al*., 2019). The Chinese version of GAD-7 has been validated (Lun *et al*., 2018). Cronbach's alpha of the GAD-7 in the current studies was 0.92 and 0.91, respectively.

The World Health Organization Well-Being Index (WHO-5; Bech, 1999) is a five-item self-reported measure of current mental well-being rated on a 6-point Likert scale (from ‘at no time’ to ‘all of the time’). The total score is multiplied by four to give the final score, with 0 indicating the worst and 100 indicating the best imaginable well-being. The WHO-5 has shown good criterion validity (sensitivity of 0.75–0.88 and specificity of 0.80–0.90 judged against a clinical diagnosis of depression) in young people (Blom *et al*., 2012). The Cantonese version of the WHO-5 has been validated (Kong *et al*., 2016). Cronbach's alpha of the WHO-5 was 0.90 and 0.88 in the two samples, respectively.

The Information subtest of the Wechsler Adult Intelligence Scale, third edition (WAIS-III; Wechsler, 1997) assesses general and semantic knowledge with 28 questions related to common facts (e.g. geography, important events) and was used to test divergent validity of the SLS-1. The Information subtest in Chinese has been validated (Yao *et al*., 2007).

The conscientiousness personality subscale of the Big Five Inventory (BFI; John and Srivastava, 1999) was also used for testing divergent validity. The subscale consists of nine items rated on a 5-point Likert scale (from ‘disagree strongly’ to ‘agree strongly’). The BFI has been validated in Hong Kong (Leung *et al*., 2013). Cronbach's alpha of the scale was 0.77 and 0.73 in the two samples, respectively.

Incremental validity of the item was further tested to determine its contribution to depressive and anxiety symptoms in addition to resilience, which has often been considered to play a protective role in the development of psychiatric disorders (Rutten *et al*., 2013). The ten-item Connor–Davidson Resilience Scale (CD-RISC-10; Connor and Davidson, 2003) was used, which consists of ten items rated on a 5-point Likert scale from ‘not true at all’ to ‘true nearly all the time’. The Chinese version of the CD-RISC-10 has been validated (Ye *et al*., 2017). Internal consistency of the scale was good, with Cronbach's alpha being 0.90 and 0.94, respectively.

To further explore the relative utility of the SLS-1, prior history of mental or neurodevelopmental disorder, distress symptoms, as well as sociodemographic variables including monthly household income and number of parents in the household, were also presented for the epidemiological sample in online Supplementary material S4.

### Statistical analysis

All statistical analyses were performed using SPSS version 25.0 (IBM SPSS Statistics, New York, United States). Descriptive statistics were independently generated for both samples.

Content validity of the SLS-1 was assessed using the item-level content validity index (I-CVI) and content validity ratio (CVR). Face validity was assessed using the computed impact score. Details of the criteria and computations are presented in online Supplementary material S2.

To improve representativeness, the raw SLS-1 score in study 1 (epidemiological sample) were first adjusted according to age and sex using data from the 2019 Hong Kong Census. To assess convergent and divergent validity, Spearman's correlation analyses were separately performed in the two youth samples between SLS-1 and other comparison measures. A correlation coefficient of 0.30 or higher was used to support convergent validity (Stinchfield *et al*., 2016). For each of the samples, two sets of hierarchical regression analysis were performed to assess the incremental validity of the SLS-1 in contributing to depressive symptoms (PHQ-9) and anxiety symptoms (GAD-7) beyond that of (i) age and gender and (ii) resilience (CD-RISC-10). A further set of correlation analysis was performed in the community youth sample to examine the association between baseline SLS-1 and long-term symptom outcomes assessed at 3, 6 and 12 months using the three DASS-21 subscales, PHQ-9 and GAD-7. Separate multiple regression analyses were performed for each of these symptom outcomes to examine the predictive utility of the SLS-1 controlling for age and gender.

The analyses were repeated in a subgroup of study 2 participants aged between 15 and 24 (online Supplementary material S3). Separate regression analyses controlling for age and gender were also performed in the epidemiological sample to consider the independent contributions of SLS-1, prior history of disorder, distress symptoms, household income and number of parents in the household on depressive and anxiety symptoms (online Supplementary material S4).

## Results

### Content and face validity of the SLS-1

Based on ratings by the 20 panel members, all members considered the SLS-1 as relevant or very relevant (I-CVI = 1) and essential (CVR = 1), with 17 items as important or very important (impact score = 3.66). A subgroup analysis including only the young laypersons (*n* = 12) revealed similar findings (I-CVI = 1; CVR = 1; impact score = 3.47), suggesting the SLS-1 had excellent content validity and good face validity.

### Study 1: SLS-1 validation in a large epidemiological youth sample

In study 1, 1445 participants provided complete data in the variables of interest and were included. Fifty-six percent (*n* = 808) aged between 20 and 25 years and 57% (*n* = 819) were female. Sixty-seven percent (*n* = 959) reported their highest level of education being secondary education and 33% (*n* = 474) reported tertiary education or above. The mean stress level measured using the SLS-1 after weighting adjustments was 5.91 (s.d. = 2.11). Using G*power software, the sample had a power of 1 for all convergent validity tests of the SLS-1.

Significant correlations were observed between scores on SLS-1 and K6 (*ρ* = 0.49), DASS-D (*ρ* = 0.37), DASS-A (*ρ* = 0.32), DASS-S (*ρ* = 0.42), PHQ-9 (*ρ* = 0.38), GAD-7 (*ρ* = 0.48) and WHO-5 (*ρ* = −0.42), all *p* < 0.001 (Table 1). Very weak associations were found between stress and scores on the Information subtest (*ρ* = 0.05, *p* = 0.040) and BFI conscientiousness (*ρ* = −0.08, *p* = 0.001). The SLS-1 was weakly correlated with CD-RISC-10 (*ρ* = −0.22, *p* < 0.001).

**Table 1.** Association between baseline scores on the SLS-1 and measures for assessing convergent and divergent validity, including K6, DASS-21 subscales, PHQ-9, GAD-7, WHO-5, as well as WAIS-III Information subtest, and BFI conscientiousness, in both the epidemiological youth sample (*n* = 1445) and community youth sample (*n* = 258)

	K6	DASS-D	DASS-A	DASS-S	PHQ-9	GAD-7	WHO-5	WAIS-III Information	BFI Conscientiousness
Baseline SLS-1 in sample 1 (*n* = 1445)	0.49***	0.37***	0.32***	0.42***	0.38***	0.48***	−0.42***	0.05*	−0.08**
Baseline SLS-1 in sample 2 (*n* = 258)	0.50***	0.47***	0.35***	0.45***	0.45***	0.53***	−0.40***	0.09	0.01

Spearman's rho coefficients (*ρ*) are presented for all correlations in both sample 1 (epidemiological youth sample) and sample 2 (community youth sample).

BFI = Big Five Inventory; DASS-A = Anxiety Subscale of the Depression Anxiety Stress Scales (DASS-21); DASS-D = Depression Subscale of the DASS-21; DASS-S = Stress Subscale of the DASS-21; GAD-7 = General Anxiety Disorder-7; K6 = 6-item Kessler Psychological Distress Scale; PHQ-9 = Patient Health Questionnaire-9; SLS-1 = Single-item Subjective Level of Stress; WAIS-III = Wechsler Adult Intelligence Scale, third edition; WHO-5 = World Health Organization Well-Being Index.

*p < 0.05, **p < 0.01, ***p < 0.001.

The additional contribution of the SLS-1 to depressive and anxiety symptoms was assessed using two separate hierarchical regression models. After controlling for age and gender in block 1, resilience in block 2 explained 15.9% of the variance in PHQ-9. The SLS-1 significantly increased the explained variance to a total of 23.5% (Δ*F* = 142.92, *p* < 0.001). Similarly, the SLS-1 significantly contributed to the variance explained for GAD-7. After accounting for age, gender and resilience, the SLS-1 explained an additional variance of 13.5% in block 3 (Δ*F* = 267.32, *p* < 0.001), with the variables altogether explaining 27.9% of the variance. Findings of the added variance of SLS-1 beyond age and gender on PHQ-9 and GAD-7 are presented alongside those of prior history of disorder, distress symptoms and other sociodemographic variables in online Supplementary material S4.

### Study 2: predictive utility of SLS-1 for 12-month outcomes

In study 2, 258 participants provided complete data and were included in the current analyses (see online Supplementary Fig. S1 for flow of participants). Forty percent (*n* = 104) aged between 20 and 25 years and 53.5% (*n* = 138) aged between 15 and 19 years. Slightly over half (56.6%, *n* = 146) were female. Fifty-six percent (*n* = 144) reported secondary education being their highest level of education, with none reporting primary education or below. The mean level of stress in this sample at baseline was 6.12 (s.d. = 1.92).

As participants were recruited as part of the larger ongoing project, follow-up assessments of a proportion of participants were not yet due and hence were not presented in the current study. Further analyses were performed in those with complete follow-up data at 3-month and 6-month (*n* = 182; 70.5%) as well as at 12-month (*n* = 84; 32.6%). No significant differences in age and gender as well as scores on both SLS-1 and comparison measures (K6, DASS-D, DASS-D, DASS-S, PHQ-9, GAD-7, WHO-5, WAIS-III Information and BFI conscientiousness) were observed between those with and without 12-month data at the time of analysis (*p* < 0.05).

#### Relationship with baseline mental health measures

Similar to study 1, the SLS-1 was significantly correlated with K6 (*ρ* = 0.50), DASS-D (*ρ* = 0.47), DASS-A (*ρ* = 0.35), DASS-S (*ρ* = 0.45), PHQ-9 (*ρ* = 0.45), GAD-7 (*ρ* = 0.53) and WHO-5 (*ρ* = −0.40), all *p* < 0.001 (Table 1). No significant associations were found between SLS-1 and the Information subtest (*ρ* = 0.09, *p* = 0.15) and BFI conscientiousness (*ρ* = 0.01, *p* = 0.87). Weak correlations were observed between stress and resilience (*ρ* = −0.20, *p* = 0.001).

In the model for PHQ-9, after controlling for age and gender, resilience in block 2 explained 10.6% of the variance. When SLS-1 was added in block 3, the variance significantly increased to 24.7% (Δ*F* = 48.16, *p* < 0.001). In the model for GAD-7, resilience explained 17.4% of the variance after controlling for age and gender, while the SLS-1 explained an additional variance of 20.2% (Δ*F* = 82.89, *p* < 0.001).

#### Associations between baseline SLS-1 and long-term symptom outcomes

Significant correlations were found between baseline SLS-1 and depressive, anxiety and stress symptoms at 3-month, 6-month and 12-month follow-up (Table 2). Separate regression models accounting for age and gender revealed that baseline SLS-1 score remained to be a significant predictor of each of the symptom outcomes at all three follow-up time points (all *p* < 0.05). The significance of findings remained unchanged in the subgroup analysis with participants aged between 15 and 24 years (online Supplementary material S3).

**Table 2.** Association between scores on the SLS-1 and DASS-21 sub-scales, PHQ-9 and GAD-7 at 3-month, 6-month and 12-month follow-up in the longitudinal community youth sample

Time point	DASS-D	DASS-A	DASS-S	PHQ-9	GAD-7
3-month follow-up (*n* = 182)	0.35***	0.34***	0.37***	0.32***	0.38***
6-month follow-up (*n* = 182)	0.26***	0.23**	0.27***	0.24**	0.30***
12-month follow-up (*n* = 84)	0.24*	0.32**	0.34**	0.24*	0.40***

DASS-A = Anxiety Subscale of the Depression Anxiety Stress Scales (DASS-21); DASS-D = Depression Subscale of the DASS-21; DASS-S = Stress Subscale of the DASS-21; GAD-7 = General Anxiety Disorder-7; PHQ-9 = Patient Health Questionnaire-9; SLS-1 = Single-item Subjective Level of Stress.

*p < 0.05, **p < 0.01, ***p < 0.001.

## Discussion

We first presented findings on the validity and utility of the SLS-1 in an epidemiological sample of young people in Hong Kong (study 1). We then applied the SLS-1 to a longitudinal cohort to examine the relationship between subjective stress and 12-month mental health outcomes (study 2). The SLS-1 had good content and face validity assessed by both experts and laypersons, as well as good convergent and divergent validity. We further showed the additional value of the SLS-1 above resilience to mental health outcomes. Importantly, in study 2, baseline SLS-1 significantly predicted 12-month depressive, anxiety and stress symptoms. These findings support the validity and predictive utility of this simple and efficient measure with potentially important research and clinical implications.

Our findings are consistent with the existing literature to show that subjective stress is a significant indicator of different mental health outcomes, including symptoms of distress, depression, anxiety and stress, and general well-being (Schneiderman *et al*., 2005). The correlations between SLS-1 and other well-established measures of symptoms are comparable to those previously reported for the validation of single-item measures (*r* = 0.36–0.50 in Littman *et al*., 2006) and support its convergent validity. The independent contributions of stress and resilience to mental health outcomes supported the potential interactions between stress and individual psychological mechanisms (Rutten *et al*., 2013). In addition, the significant relationships between baseline SLS-1 and long-term symptom outcomes further suggest its potential capability to predict the persistence of depressive symptoms beyond more transient responses to stress such as in adjustment disorder, where symptoms subside after 6 months.

We note that the support for the SLS-1 should be interpreted in the context of limitations of the two studies. Although study 1 was based on epidemiological sampling, it was a cross-sectional observation. The sample of study 2 was recruited from local youth centres and may present clinical profiles different from a general youth population. Nonetheless, our primary goal was to assess the validity and utility of the SLS-1. Attrition in the longitudinal study demands that, strictly speaking, the predictive validity is relevant only for non-dropout participants. We however observed there were no major demographic and symptom differences between the dropout and non-dropout subgroups. Overall, data from the current two studies provided initial support for the use of SLS-1 in future research. It would be worthwhile to explore its use in populations other than youth. In particular, further validation would be required in elderly and child populations. In addition, although core subjective experiences of ‘stress’ may be shared amongst different ethnic and linguistic groups, there may be local variations. Appropriate considerations of cultural adaptation, local norming and use of control groups are recommended.

As a single-item instrument, the SLS-1 inherits the same limitations as similar brief instruments (Elo *et al*., 2003; DeSalvo *et al*., 2006). Single-item tools are expected to directly relate to the underlying latent constructs without the benefit of convergence of multiple items. As such, the target construct of a global ‘subjective experience of stress’ is addressed by a direct item (‘Please indicate the level of stress which you consider you have experienced in the past one month’). Content validity would then play a relatively important role compared to internal consistency in multiple-item tools. The use of single-item measures is still in the early stages and are often used in situations where assessment burden might compromise participation and sampling, particularly in populations more difficult to engage and where help-seeking is low (e.g. young people). Such tools are not intended to replace existing established tools for clinical screening or diagnostic purposes. Rather than viewing them as mutually exclusive, the SLS-1 may capture subjective experiences of stress as a separate domain which complement information about symptomatology. In situations where more comprehensive assessments are possible, the SLS-1 may be used in combination with other symptom measures.

Previous studies have supported the use of single items as an early screening of risk (DeSalvo *et al*., 2006). The low assessment burden makes it highly suitable for inclusion as population health measures and in settings where efficiency is demanded and where resources are limited. Especially during large-scale population-level crises, brief measures with valid indicators of mental health are critical. The COVID-19 pandemic is a clear example, where large-scale data collection is only possible for when burden is kept to a minimum. The SLS-1 presented as a potentially promising option for such use.

We acknowledge that the SLS-1 focused on subjective ratings of stress, which may differ from physiological and biological measures of stress (Epel *et al*., 2018). Examining how subjective stress might complement measures such as cortisol levels and heart rate variability may provide further insights into the understanding of mechanisms underlying the multiple facets of stress. Whether the item could effectively measure stress reactivity in response to real-world stressors and suggest need for intervention may also be further explored using experience sampling methods (Myin-Germeys *et al*., 2018).

In conclusion, the SLS-1 is a valid measure of the subjective stress level that is significantly related to both concurrent and longitudinal mental health outcomes. Although more research would be needed to determine its use in other populations, the findings suggest that the SLS-1 may be used as a screening tool for early detection of individuals at higher mental health risks on a large-scale basis.

## Data Availability

The data presented in the current manuscript could be made available upon reasonable request. Enquiries may be submitted to the corresponding author.

## Supplementary material

For supplementary material accompanying this paper visit http://dx.doi.org/10.1017/S2045796021000445. click here to view supplementary material
