# Supplementary material for: Measuring subjective stress among young people in Hong Kong: validation and predictive utility of the single-item subjective level of stress (SLS-1) in epidemiological and longitudinal community samples
Source: Epidemiol Psychiatr Sci. 2021 Sep 8;30:e61. doi: 10.1017/S2045796021000445 (PMC8444151; doi:10.1017/S2045796021000445)
Supplement: Supplementary file 1 [file S2045796021000445sup001.docx]

**Supplementary Material for the Manuscript titled**

**“Measuring Subjective Stress among Young People in Hong Kong:**

**Validation and Predictive Utility of the Single-item Subjective Level of Stress (SLS-1)**

**in Epidemiological and Longitudinal Community Samples”**

Stephanie MY Wong^1^, Bess YH Lam^1^, Corine SM Wong^1^, Hattie PY Lee^1^, Gloria HY Wong^2^, Simon SY Lui^1^, KT Chan^1^, Michael TH Wong^1^, Sherry KW Chan^1,3^, WC Chang^1,3^, Edwin HM Lee^1^, YN Suen^1^, Christy LM Hui^1*^, Eric YH Chen^1,3^

^1^ Department of Psychiatry, LKS Faculty of Medicine, University of Hong Kong, Hong Kong

^2^ Department of Social Work and Social Administration, University of Hong Kong, Hong Kong

^3^ Key Laboratory of Brain and Cognitive Sciences, University of Hong Kong, Hong Kong

*Corresponding author:

Dr Christy Lai Ming Hui

(Associate Professor, Department of Psychiatry, University of Hong Kong)

Address: Department of Psychiatry, University of Hong Kong, 2/F New Clinical Building, Queen Mary Hospital, Pokfulam Road, Hong Kong

Email: [christy@lmhui.com](mailto:christy@lmhui.com)

Number: (852) 2255 3064

**Table of Content**

[Supplementary Material S1. Flow chart of participants in Study 2. 3](#_Toc71493583)

[Supplementary Material S2. Calculations of content and face validity of the SLS-1. 4](#_Toc71493584)

[Supplementary Material S3. Subgroup analysis in participants in the longitudinal community youth sample (Study 2) aged between 15 and 24 years (n=242). 5](#_Toc71493585)

[Table S1. 6](#_Toc71493586)

[Supplementary Material S4. Relationship between the SLS-1 and other related factors of depressive and anxiety symptoms and their associations with symptom outcomes. 7](#_Toc71493587)

[Table S2. 8](#_Toc71493588)

## Supplementary Material S1. Flow chart of participants in Study 2.

Figure S1 presents the flow of participants in Study 2 over the 12-month period. The flow chart shows the number of participants at the four assessment time points (baseline, 3-month and 6-month, and 12-month), including those who were not included in the current analyses for reasons of follow-up assessments not due or loss to follow-up (Figure S1).


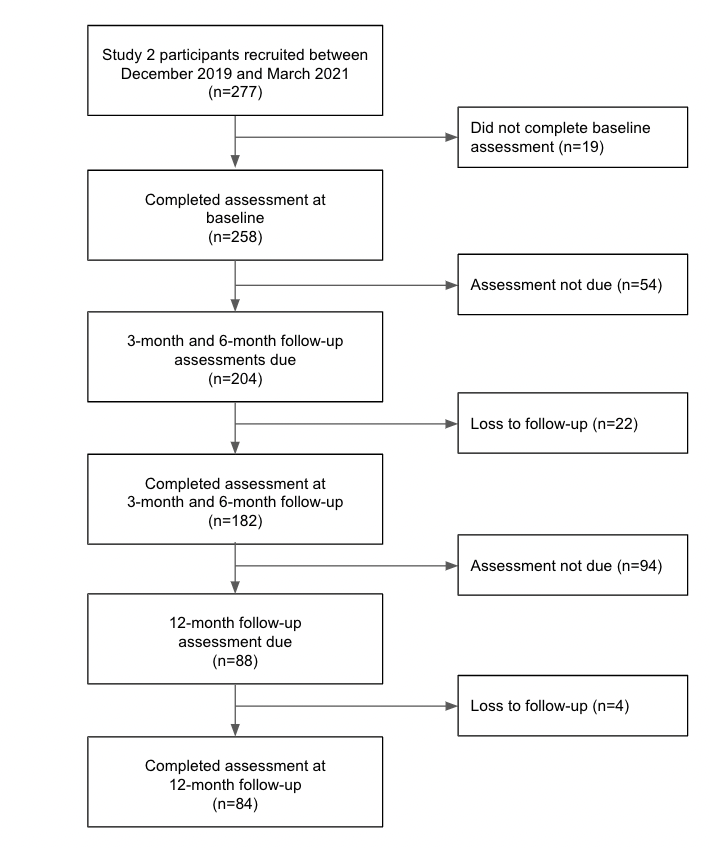


**Figure S1.** Flow chart of participants over the 12-month period in the longitudinal community study (Study 2)

## Supplementary Material S2. Calculations of content and face validity of the SLS-1.

A panel was formed with eight mental health experts (psychiatrists and senior researchers of the project team) and 12 laypersons (young people not involved in the current work) to assess the content and face validity of the SLS-1 using an anonymous online questionnaire.

Content validity of the SLS-1 using the item-level content validity index (I-CVI; Polit & Beck, 2006) for item relevance and content validity ratio (CVR; Lawshe, 1975) for item essentiality. Each panel member was first asked to rate the SLS-1 on a 4-point Likert scale from 1 (not relevant) to 4 (very relevant). The number of ratings of “relevant” or “very relevant” was divided by the number of panel members to yield the I-CVI (Polit & Beck, 2006). On a scale from 1 (not necessary) to 3 (essential), the CVR was computed using the formula (N_e_ - N/2)/(N/2), where N_e_ denotes the number of times the item was rated as “essential” (Lawshe, 1975). For item face validity, panel members were asked to rate the SLS-1 from 1 (not important) to (very important) to compute an impact score (proportion of panel members rating “important” or “very important” as multiplied by the mean level of importance; Lacasse et al., 2002). The item was recommended to be retained upon meeting the criteria of (i) I-CVI≥0.78, (Polit & Beck, 2006), (ii) CVR≥0.62 (Lawshe, 1975), and (iii) impact score≥1.5 (Lacasse et al., 2002).

## Supplementary Material S3. Subgroup analysis in participants in the longitudinal community youth sample (Study 2) aged between 15 and 24 years (n=242).

***Sample characteristic***

Ninety-four percent of participants in Study 2 were between the ages of 15 and 24 (n=242). Of this subgroup, 57% (n=138) aged between 15 and 19 years and 43% (n=104) aged between 20 and 25 years. Fifty-seven percent (n=139) were female. Regarding their highest level of education, 52.9% (n=128) reported secondary education and 47.1% (n=114) reported tertiary education. The mean level of stress in this subgroup was 6.17 (SD=1.90).

As in the analyses performed for the full Study 2 sample in the main text, further analyses were performed in participants with complete follow-up data available at 3-month and 6-month (n=173; 71.5%) as well as at 12-month (n=80; 33.1%). Similarly, no significant differences in demographic characteristics (including age and gender) and scores on the SLS-1 and other comparison measures (including K6, DASS-D, DASS-D, DASS-S, PHQ-9, GAD-7, WHO-5, WAIS-III Information, and BFI conscientiousness) were observed between those with and without 12-month follow-up data at the time of analysis (all p<0.05).

***Relationship between SLS-1 score and other mental health measures at baseline***

The relationship between SLS-1 scores and baseline mental health measures were similar to those found in Study 1 and in the full Study 2 sample.

The stress item was significantly correlated with K6 (ρ=0.50), DASS-D (ρ=0.48), DASS-A (ρ=0.37), DASS-S (ρ=0.47), PHQ-9 (ρ=0.44), GAD-7 (ρ=0.55), and WHO-5 (ρ=-0.40), all p<0.001. No significant associations were found between SLS-1 and the Information subtest (ρ=0.09, p=0.18) and BFI conscientiousness (ρ=0.01, p=0.91). Weak correlations were observed between stress and resilience (ρ=-0.22, p=0.001).

In the regression model for PHQ-9, after controlling for age and gender, resilience in Block 2 explained 13% of the variance. When stress was added in Block 3, the variance significantly increased to 27% (ΔF=46.4, p<0.001). In the model for GAD-7, resilience explained 18.3% of the variance after controlling for age and gender, while the SLS-1 explained an additional variance of 21.7% (ΔF=86.9, p<0.001).

***Associations between baseline stress level and long-term symptom outcomes***

As in findings for the full sample in Study 2, significant correlations were found in this subgroup between baseline SLS-1 and depressive, anxiety, and stress symptoms at 3-month, 6-month, and 12-month follow-up. Table S1 presents the Spearman’s correlation coefficients. Separate regression models accounting for age and gender revealed that baseline SLS-1 score remained to be a significant predictor of each of the symptom outcomes at all three follow-up time points (all p<0.05).

### Table S1.

Association between baseline SLS-1 score and DASS-21 sub-scales, PHQ-9, and GAD-7 at 3-month, 6-month, and 12-month follow-up in participants of the longitudinal community youth study aged between 15 and 24 years

| Time point | DASS-D | DASS-A | DASS-S | PHQ-9 | GAD-7 |
| --- | --- | --- | --- | --- | --- |
| 3-month follow-up (n=173) | 0.37*** | 0.35*** | 0.36*** | 0.34*** | 0.40*** |
| 6-month follow-up (n=173) | 0.30*** | 0.26*** | 0.30*** | 0.27*** | 0.33*** |
| 12-month follow-up (n=80) | 0.27* | 0.35** | 0.36** | 0.23* | 0.42*** |
| DASS-A=Anxiety subscale of the Depression Anxiety Stress Scales (DASS-21); DASS-D=Depression subscale of the DASS-21; DASS-S=Stress subscale of the DASS-21; GAD-7=General Anxiety Disorder-7; PHQ-9=Patient Health Questionnaire-9; SLS-1=Single-item Subjective Level of Stress.  *p<0.05, **p<0.01, ***p<0.001. | | | | | |

## Supplementary Material S4. Relationship between the SLS-1 and other related factors of depressive and anxiety symptoms and their associations with symptom outcomes.

In addition to the analyses presented in the main text, further analyses were performed to consider the influence of other potentially related factors of symptom outcomes, including (i) prior history of mental or neurodevelopmental disorder, (ii) monthly household income, and (iii) number of parents in the household, as well as K6 (as a key measure of distress), on depressive and anxiety symptoms in the epidemiological youth sample (sample of Study 1).

In the epidemiological sample of young people (Study 1), information on monthly household income was available from 53.5% (n=773) of the participants. The reported household income was as follows: <$10000 in 6.3% (n=49), $10,000–$29,999 in 40.2% (n=311), $30000–$59999 in 33.5% (n=259), $60000–$99999 in 13.2% (n=102), ≥$100000 in 6.7% (n=52). Seventy-one percent of the participants (n=1032) reported having two parents in the household, 21.1% (n=305) reported living with one parent, and 7.5% (n=108) lived on their own. Nine percent (n=132) reported having received a prior diagnosis of mental or neurodevelopmental disorder.

Separate hierarchical regression analyses were performed with age and gender in Block 1, and the variables of interest, including SLS-1, prior history of mental or neurodevelopmental disorder, K6, as well as monthly household income and number of parents in the household in Block 2, to determine their associations with depressive (PHQ-9) and anxiety (GAD-7) symptoms. Table S2 presents the standardised beta coefficient of the SLS-1, K6, and other sociodemographic variables in the final block of the separate hierarchical regression models (after accounting for age and gender) as well as the resulting *R*-square change after their inclusion in the model.

Higher monthly household income and number of parents in the household showed weak negative correlations with depressive symptoms (monthly household income: ρ=-0.08; number of parents in the household: ρ=-0.09), with no significant correlations observed with anxiety symptoms. Meanwhile, as expected, prior history of mental or neurodevelopmental disorder was associated with both depressive and anxiety symptoms (ρ=0.21 for both) and led to an *R*-square change of 0.05 and 0.04, respectively. As a measure of distress symptoms, K6 was strongly correlated with both depressive and anxiety symptoms (ρ=0.74 and ρ=0.78) and led to an *R*-square change of 0.55 and 0.60. The SLS-1, as a measure of subjective stress, showed to be reliably correlated with both depressive and anxiety symptom dimensions (with moderate correlation coefficients of ρ=0.36 and ρ=0.44) and led to an *R*-square change of 0.13 and 0.20. These findings provided further support for the validity of the SLS-1 as a measure of subjective stress and an indicator of other mental health outcomes.

### Table S2.

Overview of the standardised beta coefficients and *R*-square change of SLS-1, prior history of disorder, K6, and other sociodemographic variables in the separate hierarchical regression models for PHQ-9 and GAD-7, respectively.

|  | PHQ-9 | |  | GAD-7 | |
| --- | --- | --- | --- | --- | --- |
| Variables | *β* | *R*^2^ Change |  | *β* | *R*^2^ Change |
| SLS-1 | 0.36*** | 0.13*** |  | 0.44*** | 0.20*** |
| Prior history of mental or neurodevelopmental disorder | 0.21*** | 0.05*** |  | 0.21*** | 0.04*** |
| K6 | 0.74*** | 0.55*** |  | 0.78*** | 0.60*** |
| Monthly household income | -0.08* | 0.01** |  | -0.02 | 0.00 |
| Number of parents in the household | -0.09** | 0.01** |  | -0.05 | 0.00 |
| Spearman’s rho coefficients (ρ) are presented. K6=6-item Kessler Psychological Distress Scale; GAD-7=General Anxiety Disorder-7; PHQ-9=Patient Health Questionnaire-9; SLS-1=Single-item Subjective Level of Stress. *p<0.05, **p<0.01, ***p<0.001. | | | | | |

**References**

**Lacasse Y, Godbout C and Sériès F** (2002) Health-related quality of life in obstructive sleep apnoea. *The European Respiratory Journal* **19**, 499-503.

**Lawshe CH** (1975) A quantitative approach to content validity. *Personnel Psychology* **28**, 563-575.

**Polit DF and Beck CT** (2006) The content validity index: Are you sure you know what's being reported? Critique and recommendations. *Research in Nursing & Health* **29**, 489-497.
